# Identifying Novel Biomarkers and Therapeutic Targets for Endometriosis: Integrative Analysis of the Plasma Proteome and Genome

**DOI:** 10.1155/mi/6617402

**Published:** 2026-05-23

**Authors:** Jiao Zheng, Ying-Ling Yao, Xin-Zhen Chen, Li-Juan Fu, Yu-Gang Chi, Zhao-Hui Zhong, Yu-Bin Ding

**Affiliations:** ^1^ Department of Obstetrics and Gynecology, Women and Children’s Hospital of Chongqing Medical University, Chongqing, 401147, China, cqmu.edu.cn; ^2^ Department of Epidemiology, Joint International Research Laboratory of Reproduction and Development of the Ministry of Education of China, School of Public Health, Chongqing Medical University, Chongqing, 400016, China, cqmu.edu.cn; ^3^ Department of Pharmacology, Academician Workstation, Changsha Medical University, Changsha, 410219, China, csmu.edu.cn

**Keywords:** biomarkers, endometriosis, Mendelian randomization, therapeutic targets

## Abstract

**Background:**

There is currently no consensus on the etiology, pathogenesis, or treatment of endometriosis (EM). The discovery of disease‐associated plasma proteins with causal genetic evidence provides an opportunity to identify new EM biomarkers and therapeutic targets.

**Methods:**

Protein quantitative trait loci (pQTLs) were derived from plasma proteomic associations in the UK Biobank Pharma Proteomics Project (UKB‐PPP). Genetic associations with EM were obtained from the FinnGen cohort. The associations between proteins and the risk of EM were estimated by cis‐Mendelian randomization (cis‐MR) and validated using the GWAS catalog dataset of EM. Colocalization, protein–protein interaction (PPI) analysis, functional enrichment analysis, transcriptome differential expression gene (DEG) analysis and druggability evaluation were further performed to explore potential biomarkers and therapeutic targets for EM.

**Results:**

Overall, genetically predicted levels of 23 plasma proteins were associated with EM risk, with five proteins validated via replication analysis (ALPI, KHK, HSPG2, STXBP1, and POLR2F). Lower levels of genetically predicted ALPI (odds ratio [OR]: 0.89, 95% confidence interval [CI] 0.83–0.95), HSPG2 (OR: 0.81, 95% CI 0.75–0.88), POLR2F (OR: 0.51, 95% CI 0.36–0.73), and STXBP1 (OR: 0.75, 95% CI 0.64–0.86) were associated with an increased risk of EM. Elevated levels of KHK (OR: 1.09, 95% CI 1.05–1.13) were associated with an increased risk of EM. We also identified ALPI, KHK, HSPG2, STXBP1, and POLR2F as potential drug targets and biomarkers for EM.

**Conclusion:**

A series of comprehensive analyses emphasized the potential role of ALPI, KHK, HSPG2, STXBP1, and POLR2F in EM and suggested that these genes could be developed into exact biomarkers and therapeutic targets for this condition in future research.

## 1. Introduction

Endometriosis (EM) is a chronic inflammatory disease characterized by the presence of tissue outside the uterus that resembles the endometrium, which causes pelvic pain and infertility [1]. Affecting ~176 million women of childbearing age globally, EM poses a significant socioeconomic burden and severely impacts the quality of life [2]. Despite its prevalence, the mechanisms underlying EM development remain unclear, and diagnosis is often delayed due to the lack of specific symptoms and readily accessible biomarkers [3].

Efforts have been made to identify peripheral biomarkers for EM, including immunological, genetic, hormonal, and biochemical candidates such as Cancer Antigen 125 (CA‐125), cytokines, microRNAs, and autoantibodies [4–7]. However, many of these markers lack specificity or are elevated in other gynecological or inflammatory conditions, limiting their diagnostic accuracy [5, 8]. For example, CA‐125 is commonly elevated not only in EM but also in ovarian cancer and menstruation, which limits its diagnostic utility [9]. Furthermore, their sensitivity is often suboptimal, and no single marker has yet demonstrated sufficient performance for routine clinical application (PMID: 34048704). In addition to their diagnostic limitations, many of these molecular targets also fail to translate into effective therapies. Nevertheless, emerging evidence suggests that several inflammatory and endocrine‐related molecules may serve as promising therapeutic candidates for EM. These include pro‐inflammatory cytokines such as interleukin‐6 (IL‐6), interleukin‐8 (IL‐8), and tumor necrosis factor‐alpha (TNF‐α); angiogenic growth factors such as vascular endothelial growth factor (VEGF) and transforming growth factor‐beta (TGF‐β); and the steroidogenic enzyme aromatase [9–12]. These molecules are thought to contribute to the chronic inflammatory and estrogenic microenvironment that sustains ectopic lesion survival and progression.

Mendelian randomization (MR) uses genetic variation as the instrumental variable (IV) for exposure, making MR less susceptible to confounding factors and enhancing causal inference, and MR is increasingly becoming a standard tool for identifying new drug targets [13]. Few MR studies have integrated GWAS and protein quantitative trait loci (pQTLs) data [14, 15], specifically regarding EM. A previous MR analysis examined the causal relationship between the whole genome and EM and revealed that proteins such as IMMT and KLF12 were positively associated with the risk of EM [16]. A reliable causal effect of the genetic prediction of plasma ADAMTS13 levels on decreased EM risk has also been reported [17]. However, there is a critical need to systematically and comprehensively explore potential biomarkers and therapeutic targets for EM. Recent advances in plasma proteomics, exemplified by a landmark study that characterized the plasma proteomic profiles of 54,219 participants and comprehensively mapped pQTLs for 2923 proteins from the UK Biobank, have significantly contributed to identifying potential protein biomarkers and therapeutic targets for various diseases, including potentially EM [18]. This extensive dataset provides a valuable resource for understanding the molecular underpinnings of EM and identifying novel therapeutic targets.

Hence, the present study conducted a proteome‐wide MR analysis using human plasma proteome and genome data to systematically identify proteins associated with EM risk and explore their potential as therapeutic targets for this condition.

## 2. Methods

### 2.1. Study Design

**Figure 1:**
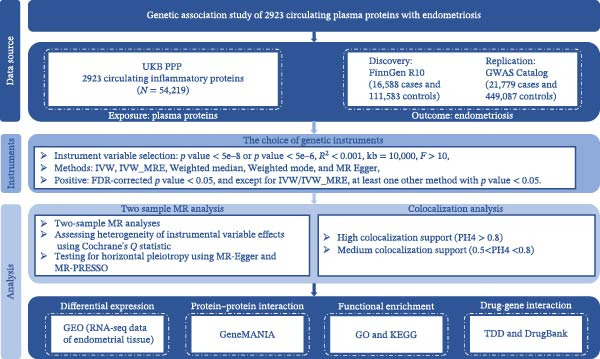
Study design flowchart based on MR for evaluating the effect of the plasma proteome on EM. UKB‐PPP, UK Biobank Pharma Proteomics Project; GWAS, genome‐wide association study; MR, Mendelian randomization; IVW, inverse variance weighted; IVW‐MRE, inverse variance weighted (multiplicative random effects).

Figure 1 illustrates the schematic study design of MR approaches. In this study, we initially conducted proteome‐wide MR and colocalization analyses to identify plasma proteins associated with EM.

### 2.2. Study Exposures

UK Biobank is a large‐scale biomedical database and research resource containing genetic, lifestyle, and health information from half a million UK participants. The exposures used in this study were genetically predicted plasma proteins from the UK Biobank Pharma Proteomics Project (UKB‐PPP) [18]. Details of the study are presented in Supporting Information 1: Table S1. Cis‐pQTLs are defined when the main SNP in the region is located within 1Mb of the transcription start site of the protein‐coding gene, while pQTLs located outside this region are categorized as trans‐pQTLs. Given the greater prior probability of specific biological effects associated with cis‐pQTLs than trans‐pQTLs, we employed cis‐pQTLs as a genetic instrument for MR analysis to systematically evaluate the causal effects of plasma proteins on EM. We used the following criteria for selecting instruments and proteins: (i) We first extracted the conventional genome‐wide significance *p* value threshold for instrument selection to *p*  < 5e–8. However, considering the exclusion of 591 plasma proteins out of 2923 plasma proteins and 299 SNPs that did not exceed three significantly correlated SNPs at the genome‐wide significance level, we relaxed the *p* value threshold for instrument selection to *p*  < 5e–6. To balance proteomic coverage and instrument strength, the relaxed threshold was applied only at the screening stage, while instrument validity was ensured through cis‐restriction, multiple independent SNPs, and *F*‐statistic evaluation. (ii) Linkage disequilibrium (LD) clustering was performed with *r* ^2^ > 0.001 and upstream/downstream distances <10000 kb as thresholds to identify independent pQTLs for each protein. (iii) *F* is used to estimate the strength of genetic tools (*F* = (beta/se) ^ 2) [19]. An *F* value above 10 was considered high instrument strength [20]. Finally, in the discovery cohort, we analyzed the relationships between 2332 proteins and EM at a threshold of *p*  < 5e–8 and between 904 proteins and EM at a threshold of *p*  < 5e–6. Each protein included in this analysis was instrumented by at least two independent SNPs to ensure the robustness of the MR estimates.

### 2.3. Study Outcomes

The FinnGen study is a large‐scale genomics initiative that has analyzed over 500,000 Finnish biobank samples and correlated genetic variation with health data to understand disease mechanisms and predispositions. The project is a collaboration between research organizations and biobanks within Finland and international industry partners. In the primary MR analysis, we obtained GWAS data for EM from the Finnish biobank to investigate EM‐associated alterations in the proteome. This dataset included 16,588 EM patients and 111,583 controls. Additionally, for external validation of the significant proteins identified in the primary analysis, we obtained summary statistics from the GWAS Catalog for EM, which included 21,779 EM patients and 449,087 controls. Summary statistics were downloaded from the NHGRI‐EBI GWAS Catalog [21] on 01/23/2024 for study GCST90205183 [22]. Supporting Information 1: Table S1 lists the sources and corresponding information of all the aggregated statistical datasets used in this study.

### 2.4. MR Analyses

MR analysis is based on the SNP‐specific Wald ratio method to evaluate the causal effect of proteins on EM, that is, the effect sizes of IVs on outcomes divided by those on exposure. The inverse variance‐weighted (IVW) method is reported to be slightly more powerful than other methods under certain conditions [23]. For proteins with 2–3 SNPs, the IVW method was primarily used for analysis. For proteins with more than three SNPs, the inverse variance‐weighted (multiplicative random effects) method is mainly employed [24, 25]. To verify the reliability of the analytical MR results, additional analyses were performed, including weighted median, MR‐Egger, and weighted mode analyses. The weighted median provides a consistent estimate of causal effects if at least 50% of the weight comes from valid IVs [23]. MR‐Egger was used to detect and correct for bias caused by directional pleiotropy. If pleiotropic effects did not cancel out (*p*‐pleiotropy < 0.05), the IVW estimate could exhibit significant bias [23, 26]. Finally, the weighted mode method is based on the clusters with the largest number of SNPs, deriving a single causal effect estimate from multiple genetic instruments [27]. FDR correction for multiple testing was conducted to correct *p* values, and a *p* value less than 0.05 was considered strong evidence of a causal association.

### 2.5. Sensitivity Analysis for MR

The heterogeneity of the genetic variants was tested based on Q statistics [28]. MR‐Egger regression was employed to examine the mean pleiotropic effect of all IVs. The global MR pleiotropy residual sum and outlier (MR‐PRESSO) (https://github.com/rondolab/MR-PRESSO/) test was introduced to explore the possible outlier SNPs and detect the presence of horizontal pleiotropy [29]. Additionally, the leave‐one‐out method sequentially removes each SNP to observe any significant changes in the effect value, thereby testing whether the research conclusion is heavily influenced by a single SNP and ensuring its robustness [30]. The MR analyses were conducted using the TwoSampleMR package (version 0.5.6) [31].

### 2.6. Colocalization Analysis

The results that met the multiple‐testing threshold in the MR analysis were further evaluated using Bayesian colocalization analysis to estimate the posterior probability that each genomic locus contains a single variant affecting both the protein and the EM‐related trait rather than the variant being coincidentally shared due to LD [32]. For this analysis, we utilized the coloc package (https://github.com/ chr1swallace/coloc), which employs Bayesian methods to estimate the posterior probability of a shared causal variant between two traits. Under the standard assumption of a single causal variant per locus, five hypotheses (H0–H4) were evaluated: H_0_, proposing the lack of causal variants for both traits; H_1_, positing the existence of a causal variant for trait 1; H_2_, suggesting a causal variant for trait 2; H_3_, postulating two distinct causal variants for traits 1 and 2; and H_4_, proposing a shared causal variant between the two traits [33]. A posterior probability greater than 0.8 for hypothesis H_4_ was defined as strong evidence of colocalization.

### 2.7. Protein–Protein Interaction (PPI) and Functional Enrichment Analysis

To investigate the relationships among the MR candidate proteins, PPI networks were constructed using a search tool for the retrieval of interacting genes (GeneMANIA, https://genemania.org/). This online database provides information on gene interactions through the use of PPI networks, coexpression patterns, and genetic connections. Furthermore, Gene Ontology (GO) and Kyoto Encyclopedia of Genes and Genomes (KEGG) pathway analyses were performed to explore the potentially enriched pathways associated with these proteins [34]. *p* < 0.05 was set as the cutoff criterion for significant enrichment.

### 2.8. Transcriptome Differential Expression Gene (DEG) Analysis

To explore differential expression between disease and control groups, we conducted transcriptome analysis using a series of matrix files from GSE7305, GSE11691, GSE25628, and GSE23339. We extracted expression profiles for 73 samples, with 37 normal and 36 disease samples. Details are given in Supporting Information 1: Table S9. We merged these datasets using the R package SilicoMerging and removed batch effects with Johnson et al.’s [35] method, resulting in a final matrix (Supporting Information 1: Table S10). The data used in this study are publicly available from the GEO database (https://www.ncbi.nlm.nih.gov/geo/). Here, we used the R software package limma (version 3.40.6) for differential expression analysis to obtain DEGs between different comparison groups and control groups [36]. Volcano plots and heatmaps were used to visually depict the distribution and expression of DEGs. DEGs were identified with the criteria of (|log_2_FC| > 1.5) and _*p*FDR_ < 0.05. Additionally, Violin plots were used to illustrate differences in gene expression levels between the case and control groups. This analysis was intended to provide complementary transcriptomic context rather than direct validation of plasma proteomic MR findings.

### 2.9. Identification of Druggable Proteins

We further evaluated whether the identified proteins could serve as potential therapeutic targets by investigating their interactions with drugs using the Therapeutic Target Database (TTD; https://db.idrblab.net/ttd/) and DrugBank databases [37], which prioritized potential drug targets by integrating information from drug‐gene interactions, gene function, and text mining. For proteins identified in drug databases, information on drug names and the development process of drugs that target identified proteins was documented. To assess potential druggability, we classified these proteins into four categories: (1) approved, (2) investigation, (3) experimental/clinical trial target, and (4) Illicit and/or withdrawn.

## 3. Results

### 3.1. Proteome‐Wide MR Analysis

**Figure 2:**
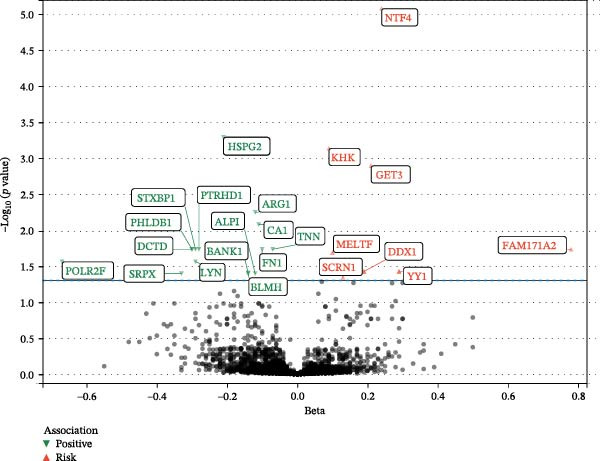
Volcano plot of MR analysis. Each point in the figure represents the MR analysis result of a protein and EM. The horizontal axis represents the beta value, and the vertical axis represents the statistical significance, which is the reciprocal of the *p* value (−log_10_ *p* value). The red upper triangle indicates a positive correlation between proteins and volcanic islands, while the green lower triangle indicates a negative correlation (*p*  < 0.05). Gray represents results that did not reach statistical significance. MR, Mendelian randomization; EM, endometriosis.

Using the fixed effects IVW method or the IVW (multiplicative random effects) method, a total of 23 proteins were significantly associated with EM risk after FDR correction (*p*  < 0.05) (Figure 2) (Supporting Information 1: Table S2). The 189 SNP genetic instruments for 23 proteins had an average *F* statistic of 174.3 (*F* > 10, ranging from 20.99 to 4475.84), which indicated the absence of weak instruments. Genetically predicted higher levels of FAM171A2, GET3, KHK, MELTF, NTF4, SCRN1, YY1, and DDX1 were associated with an increased risk of EM, while genetically predicted higher levels of the other 15 proteins (ALPI, ARG1, BANK1, BLMH, CA1, DCTD, FN1, HSPG2, LYN, PHLDB1, POLR2F, PTRHD1, SRPX, STXBP1, and TNN) were associated with a reduced risk of EM (Supporting Information 1: Table S2). All the results of the discovery queue of proteome‐wide MR are shown in Supporting Information 1: Table S3.

**Figure 3:**
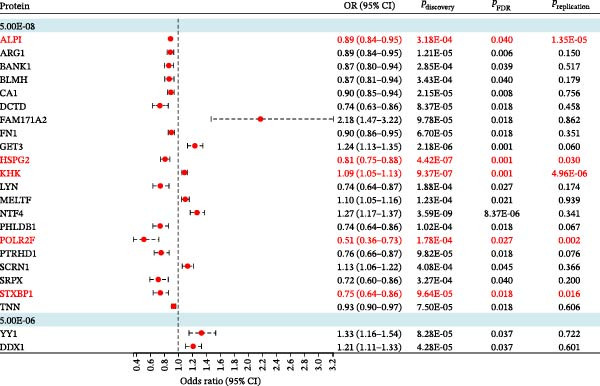
Casual effects of MR analysis between 23 identified proteins and EM in the discovery cohort. MR, Mendelian randomization; EM, endometriosis, discovery dataset: UK Biobank; replication datasets: GWAS catalog.

In the replication stage, five proteins were successfully validated in the GWAS Catalog dataset (*p* < 0.05) (Figure 3) (Supporting Information 1: Table S4). The odds ratio (OR, 95% confidence interval, CI) of EM per SD increase in genetically predicted levels of protein was 0.93 (0.90–0.96) for ALPI, 0.90 (0.83–0.99) for HSPG2, 0.61 (0.44–0.83) for POLR2F, 0.76 (0.60–0.95) for STXBP1, and 1.06 (1.04–1.09) for KHK. All the results of the replication of proteome‐wide MR are shown in Supporting Information 1: Table S5. The scatter plots show a consistent trend. (Supporting Information 2: Figure S1).

### 3.2. Sensitivity Analysis for MR

We used funnel plots to visually assess pleiotropy (Supporting Information 3: Figure S2). Although the asymmetry of the funnel plot is indicative of directional horizontal pleiotropy, it is difficult to assess the symmetry of the funnel plot because of the small number of genetic variants. Moreover, there was no heterogeneity (_*p*heterogeneity_ > 0.05) or pleiotropy (_*p*pleiotropy_ > 0.05 and _*p*MR-presso_ > 0.05) (Table 1; Supporting Information 1: Table S6). To identify potentially strongly influential SNPs on the overall estimate, we used the leave‐one‐out method, revealing no substantial difference across the estimated causal effects (Supporting Information 4: Figure S3). The forest plot shows individual SNP‐level and pooled estimates, illustrating the effect of plasma proteins on EM (Supporting Information 5: Figure S4.

**Table 1:** Sensitivity analysis of five candidate proteins determined by Mendelian randomization.

Plasma protein	Nsnp	Pleiotropy		MR‐PRESSO		Heterogeneity analyses		
		Egger‐intercept	*p*_pleiotropy_	Global test *p*	Correct *p*	Method	*Q*	*Q p* value
ALPI	16	−0.009	0.464	0.076	NA	MR‐Egger	22.45	0.070
						IVW	23.36	0.077
HSPG2	8	0.013	0.181	0.673	NA	MR‐Egger	1.66	0.949
						IVW	3.95	0.786
KHK	8	−0.006	0.561	0.452	NA	MR‐Egger	1.07	0.899
						IVW	1.47	0.92
POLR2F	2	NA	NA	NA	NA	IVW	0.06	0.81
STXBP1	7	0.017	0.477	0.668	NA	MR‐Egger	3.94	0.56
						IVW	4.53	0.61

*Note:* ALPI, intestinal‐type alkaline phosphatase; HSPG2, basement membrane‐specific heparan sulfate proteoglycan core protein; KHK, ketohexokinase; POLR2F, DNA‐directed RNA polymerases I, II, and III subunit RPABC2; STXBP1, syntaxin‐binding protein 1; IVW, inverse variance weighted.

### 3.3. Colocalization Analysis

Among the five potential candidate proteins identified by proteome‐wide MR, there was no strong evidence of genetic colocalization (_*p*H4_ < 0.80) under different priors and windows (Table 2). Notably, a limitation of Bayesian colocalization is the assumption of a single shared common causal SNP. However, in reality, genetic loci may contain several causal SNPs [38].

**Table 2:** Colocalization results.

Plasma protein	Full summary data available	Colocalization						Robust colocalization evidence
		Nsnp	*p*_H0_	*p*_H1_	*p*_H2_	*p*_H3_	*p*_H4_	
ALPI	Yes	7494	0.711	0.145	0.116	0.024	0.004	No
HSPG2	Yes	33,350	8.66E‐114	1.35E‐96	6.44E‐18	1.00	3.84E‐17	No
KHK	Yes	4501	1.01E‐311	1.43E‐312	0.854	0.121	0.025	No
POLR2F	Yes	6599	0.666	0.192	0.107	0.031	0.004	No
STXBP1	Yes	5149	8.75E‐8	2.56E‐8	0.744	0.218	0.039	No

### 3.4. Investigating PPI and Enriched Pathways of the MR‐Candidate Proteins

**Figure 4:**
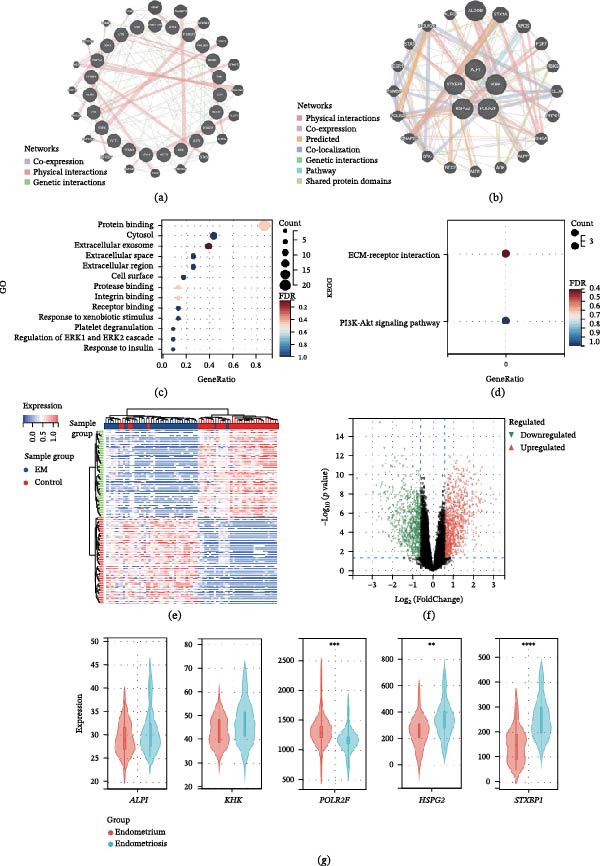
The subsequent analysis of Mendelian randomization results on the associations between plasma proteins and the endometriosis. (a,b) Networks of identified proteins associated with endometriosis. The network prediction was based on an online tool: Gene MANIA (http://www.genemania.org). (c) Gene Ontology (GO) enrichment analysis of DEGs. (d) Kyoto Encyclopedia of Genes and Genomes (KEGG) enrichment analysis of DEGs. (e) Heatmap shows DEGs of EM. (f) The volcano plot shows DEGs of EM. (g) Violin plot of differences in the expression levels of five candidate plasma proteins of EM,  ^∗∗∗^ represents a statistically significant difference (*p*  < 0.05).

To understand the potential relationships between candidate proteins and their enrichment functions and to further elucidate the pathogenesis of EM, we conducted PPI and pathway analyses to explore the associations among the identified proteins. Using the GeneMANIA online tool, we constructed a PPI network, as depicted in Figure 4a,b, in which the identified EM‐related proteins formed a network characterized by physical interactions and coexpression. Moreover, the five plasma proteins identified by the replication queue within GeneMANIA were analyzed using pathway analysis tools. We found that these tightly interacting genes are mainly related to intercellular signal transduction and material transport, and they work together to regulate the function of the nervous system, including processes such as vesicle fusion to the plasma membrane, synaptic vesicle exocytosis, neurotransmitter secretion, vesicle fusion, and neurotransmitter regulation.

KEGG pathway enrichment analysis identified 23 candidate proteins in the discovery cohort, and two pathways were enriched (Figure 4d). The results indicate that TNN, FN1, and HSPG2 are mainly involved in extracellular matrix (ECM)‐receptor interactions, while NTF4, TNN, and FN1 are mainly associated with the PI3K‐Akt signaling pathway. In the GO pathway enrichment analysis, pathways such as protein binding, cytosol, extracellular exosome, extracellular space, and cell surface were enriched for the cis‐Mendelian randomization (cis‐MR) candidate proteins. Furthermore, pathways related to protease binding, integrin binding, integrin binding, response to xenobiotic stimulus, response to xenobiotic stimulus, platelet degranulation, and regulation of the ERK1 and ERK2 cascades were also enriched for the cis‐MR candidate proteins (Figure 4c).

### 3.5. Transcriptome DEG Analysis

We used the limma software package to analyze the expression of five candidate protein‐related genes in EM and found that 1828 genes had DEGs. Detailed information on all DEGs is provided in Supporting Information 1: Table S7 and Figure 4e,f. Among the five candidate genes, POLR2F was significantly downregulated (logFC = −0.20, adjusted *p* < 0.05), while STXBP1 (logFC = 0.89,adjusted *p* < 0.001) and HSPG2 (logFC = 0.47, adjusted  *p* < 0.05) were upregulated, while KHK and ALPI showed no significant expression changes (adjusted  *p* > 0.05) (Figure 4g). Notably, transcriptomic validation reflects tissue‐level mRNA expression and may not directly correspond to genetically predicted circulating protein effects.

### 3.6. Druggability of the Identified Proteins

We searched for five circulating proteins identified as potential biomarkers in MR analysis in the drug database but did not find them to be drug targets for EM (Supporting Information 1: Table S8). Terpinen‐4‐ol, a drug targeting ALPI, has been developed as a new therapy for combating melanoma, and another drug, vicagrel, is also being tested in antiplatelet therapy experiments. The drug palifenin, which targets HSPG2, has been approved for the treatment of oral mucositis associated with, and efanesoctocog alfa is indicated for use in adults and children with hemophilia A (congenital factor VIII deficiency) for routine prophylaxis to reduce the frequency of bleeding episodes. There is no information available regarding POLR2F, KHK, and STXBP1 in the drug database we searched.

## 4. Discussion

In this study, we conducted a comprehensive proteome‐wide MR analysis to explore the causal associations between plasma proteins and the risk of EM. Leveraging genetic instruments for 2332 proteins at the stringent genome‐wide significance threshold (*p* < 5e–8) and 904 proteins at a more relaxed threshold (*p* < 5e–6), we systematically evaluated 2923 proteins in total. The discovery stage identified 23 candidate proteins, among which genetically predicted higher levels of 8 proteins and lower levels of 15 proteins were associated with increased EM susceptibility. Replication analyses further confirmed robust associations for five proteins—ALPI, HSPG2, POLR2F, KHK, and STXBP1. Functional characterization through pathway enrichment, PPI, and transcriptomic integration highlighted their biological plausibility, with key involvement in ECM–receptor interaction, PI3K–Akt signaling, synaptic vesicle exocytosis, and neurotransmitter regulation. The lack of differential expression for KHK and ALPI in tissue transcriptomic analyses likely reflects distinct regulatory layers rather than inconsistency with plasma proteomic MR findings. Importantly, while ALPI and HSPG2 have been previously studied as therapeutic targets in cardiovascular diseases and cancers, POLR2F, KHK, and STXBP1 represent novel proteins without prior drug annotations, thereby pointing to unexplored therapeutic opportunities.

Compared with a recent proteome‐wide MR study of EM [39], which prioritized EPHB4, FSHB, RSPO3, and SEZ6L2, our analysis yielded a distinct set of candidate proteins. This divergence can largely be attributed to methodological differences in instrument selection. The prior study limited instruments to only the top SNP per cis region (±1Mb from the transcription start site), which constrained the analysis to 1632 proteins. In contrast, our approach required at least two independent SNPs per protein after stringent LD clumping and allowed multiple instruments per protein, thereby increasing robustness and statistical power. This broader strategy expanded the analytic coverage and enabled us to detect novel candidates, including POLR2F and STXBP1, that were not captured previously. Taken together, these differences demonstrate that our study is not merely a replication but a substantive extension of earlier work, offering complementary insights into the proteomic architecture of EM and identifying promising new directions for biomarker discovery and therapeutic development.

### 4.1. ALPI: A Potential Target for Inflammation

ALPI, a brush border enzyme involved in the hydrolyzing of phosphate compounds, plays a role in gut mucosal defense and the detoxification of lipopolysaccharides [40, 41]. Although no studies have directly linked ALPI to EM, its role in mucosal immunity and inflammation may have implications for the disease. In murine models of EM, antiplatelet therapy significantly reduced markers of angiogenesis, inflammation, and fibrosis [42]. Ongoing clinical trials of vicagrel, an ALPI‐t argeting drug in the context of antiplatelet therapy [43], suggest translational potential. Furthermore, ALPI helps regulate intestinal inflammation and maintain gut barrier integrity, which is crucial since dysregulated inflammation and increased intestinal permeability are associated with EM [44, 45]. Therefore, alterations in ALPI levels may affect local immune responses, potentially influencing the development and maintenance of endometriotic lesions. Additionally, ALPI interactions with the gut microbiome may further modulate systemic inflammation, contributing to disease progression [46]. Natural compounds such as terpinen‐4‐ol, which targets ALPI and possesses antioxidant and anti‐inflammatory properties, which targets ALPI and possesses antioxidant and anti‐inflammatory properties [47, 48], offer further therapeutic possibilities.

### 4.2. HSPG2: Modulator of Vascular Pathology

HSPG2, a crucial component of the basement membrane, plays essential roles in angiogenesis and regulating the blood vessel response to injury. Research has confirmed that Notch signaling regulates the normalization of vascular structure and function by regulating the expression of the downstream gene HSPG2 [49]. Bleeding and subsequent tissue repair are prominent features of endometrial lesions [50], and the role of HSPG2 in maintaining vascular integrity and promoting angiogenesis suggests that it could be critical in forming and maintaining endometriotic lesions, which are characterized by aberrant blood vessel growth and increased permeability. Dysregulation of HSPG2 could contribute to the pathological angiogenesis observed in EM, thereby influencing the chronic inflammation and pain associated with the disease. Efanesoctocog alpha, a drug targeting HSPG2, is currently used for the management of bleeding in hemophilia patients and promotes fibrin clot formation and reduces platelet accumulation [51]. Its role in normalizing angiogenic responses raises the possibility of repurposing this agent to mitigate vascular abnormalities in EM.

### 4.3. POLR2F: A Transcriptional Regulator With Immuno‐Neurological Implications

POLR2F, a subunit of RNA polymerase II, is crucial for transcriptional regulation, influencing gene expression essential for cellular functions, including inflammation and tissue remodeling [52]. EM, characterized by the ectopic growth of endometrial‐like tissue outside the uterus, is associated with chronic pelvic pain, infertility, and systemic inflammation, reflecting dysregulated molecular pathways [53]. Elevated POLR2F levels may correlate with increased transcriptional activity driven by inflammatory mediators and growth factors implicated in the establishment and progression of endometrial lesions. POLR2F is also associated with Waldenburg syndrome, peripheral demyelinating neuropathy, central myelination disorders, and other psychiatric disorders, underscoring its potential relevance in the nervous and immune systems [54]. These observations position POLR2F as a promising molecular marker of disease activity and severity and a potential candidate for targeted therapies in EM.

### 4.4. KHK: Linking Fructose Metabolism and Hormonal Inflammation

KHK, also known as fructokinase, is pivotal in fructose metabolism and converts fructose to fructose‐1‐phosphate, which can lead to fat synthesis and accumulation. Excessive KHK activity promotes metabolic disorders and inflammation [55], which may exacerbate EM. The increased fat accumulation driven by KHK can result in systemic insulin resistance and other metabolic disturbances, worsening EM symptoms [8, 44]. Additionally, metabolic byproducts of fructose metabolism, such as uric acid, can activate inflammatory pathways, contributing to the chronic inflammation characteristic of EM [56]. Moreover, adipose tissue acts as an endocrine organ, and increased fat accumulation can alter the hormonal milieu, potentially affecting estrogen levels, which play a crucial role in EM [57]. This hormonal imbalance, coupled with the proinflammatory state induced by KHK activity, may exacerbate the growth and maintenance of the endometriotic tissue, as well as the severity of pain and other symptoms. Thus, KHK represents a potential biomarker for metabolic‐endocrine dysfunction in EM and may serve as a novel therapeutic target for disrupting this pathological loop.

### 4.5. STXBP1: A Link Between Neurotransmission and Chronic Pain

STXBP1 encodes a syntaxin‐binding protein that plays a critical role in the release of neurotransmitters by regulating syntaxin, a transmembrane attachment protein receptor [58]. This protein has been implicated in various conditions, including developmental and epileptic encephalopathy, through its involvement in pathways such as neurotransmitter release and energy metabolism integration [59, 60]. Chronic pain and neuroinflammation are defining features of EM [61]. We propose that dysregulation of STXBP1 may underlie the chronic pain phenotype observed in a subset of EM patients, particularly through altered neurotransmitter dynamics and central sensitization. Although STXBP1 has not been previously studied in the context of EM, it could represent an important link between neuroinflammation and symptom severity. Further investigation may elucidate whether STXBP1‐targeted strategies can alleviate pain or identify patient subgroups with neurogenic disease profiles.

The strength of this study lies in our two‐sample proteomic MR design, utilizing a large sample size to systematically examine 2923 plasma proteins and their associations with EM risk, minimizing bias from confounding and reverse causality, thereby improving causal inference. The consistency of the results across multiple analyses confirmed the robustness of our findings. Notably, we identified five novel biomarkers and therapeutic targets (ALPI, HSPG2, POLR2F, KHK, and STXBP1) previously unassociated with EM. To minimize horizontal pleiotropy, we used plasma protein cis‐pQTLs as instruments. PPI and pathway analysis further validated candidate proteins’ connections, and druggability evaluation provided insights into their pathogenic effects. Additionally, analyzing individuals of European ancestry minimized the population stratification bias. This comprehensive approach offers valuable insights into EM pathogenesis, highlighting new biomarkers and therapeutic targets for future research and clinical applications.

Some limitations of our analysis are worth noting. First, although Bayesian colocalization analyses were performed to assess whether shared causal variants underlie both plasma protein levels and EM risk, none of the identified associations reached the conventional threshold for strong colocalization. This finding should be interpreted with caution as standard colocalization approaches assume a single causal variant per locus—an assumption that may not fully capture the genetic architecture of complex traits such as EM or plasma protein regulation. Importantly, the absence of strong colocalization does not preclude a causal relationship supported by robust MR analyses with strong instruments and consistent sensitivity results. Second, the current analysis was restricted to European populations. The generalization of these findings to other ancestries needs to be further confirmed. Third, our study focuses on plasma protein levels, which may not fully capture the endometrial tissue directly involved in the pathogenesis of EM. However, using cis‐pQTLs as instruments could decrease the risk of horizontal pleiotropy. Finally, while our study identified promising therapeutic targets, the lack of existing drug information for some proteins highlights the need for more extensive pharmacological research to fully explore the therapeutic potential of these targets.

## 5. Conclusions

In conclusion, our comprehensive analyses highlight the potential role of five plasma proteins—ALPI KHK, HSPG2, STXBP1, and POLR2F—in the risk and pathogenesis of EM. These proteins are promising targets for developing screening biomarkers and therapeutic drugs for EM. Our findings offer new avenues for the early diagnosis and treatment of EM. Future research should focus on further validating these targets and exploring their mechanisms to develop effective interventions for EM.

**Nomenclature**
DEGs: Differentially expressed genes
EM: Endometriosis
FDR: False discovery rate
GEO: Gene expression omnibus
GO: Gene ontology
GWASs: Genome‐wide association studies
HEIDI: Heterogeneity in dependent instruments
IVs: Instrumental variables
IVW: Inverse variance weighted
IVW‐MRE: Inverse variance weighted (multiplicative random effects)
KEGG: Kyoto Encyclopedia of Genes and Genomes
LD: Linkage disequilibrium
MR: Mendelian randomization
PPI: Protein‐protein interaction
pQTLs: Protein quantitative trait loci
SD: Standard deviation
SNP: Single nucleotide polymorphism
TTD: Therapeutic target database
UKB‐PPP: UK Biobank Pharma Proteomics Project.

## Author Contributions

**Jiao Zheng**: writing – original draft, software, methodology, formal analysis, visualization. **Ying-Ling Yao**: writing – original draft, methodology, formal analysis. **Xin-Zhen Chen and Yu-Gang Chi**: formal analysis, data curation. **Li-Juan Fu**: formal analysis, visualization. **Zhao-Hui Zhong**: writing – review and editing. **Yu-Bin Ding**: writing – review and editing, validation, supervision.

## Funding

This work was supported by the National Key Research and Development Program of China (Grant 2023YFC2705900) and an Open Research Grant from the Key Laboratory of Perinatal Medicine, Chongqing Health Commission (Grant 2024KFKT03).

## Disclosure

All authors commented on previous versions of the manuscript and approved the final version.

## Conflicts of Interest

The authors declare no conflicts of interest.

## Supporting Information

Additional supporting information can be found online in the Supporting Information section.

## Supporting information


**Supporting Information 1** Table S1: Summary of outcome and exposure datasets used in the current study. Table S2: Results of 23 proteome‐wide Mendelian randomization identified proteins for endometriosis risk. Table S3: All results of proteome‐wide Mendelian randomization for endometriosis risk using only cis‐pQTLs. Table S4: Mendelian randomization results were determined for 23 proteins at risk of endometriosis in the replication cohort. Table S5: All results of proteome‐wide Mendelian randomization for endometriosis risk using cis‐pQTLs in the replication cohort. Table S6: Sensitivity analysis of Mendelian randomization results for proteins within 23 proteomic ranges that determine the risk of endometriosis. Table S7: All transcriptome differential gene information. Table S8: Druggability of proteins potentially causally associated with endometriosis. Table S9: Benchmark datasets retrieved from the GEO database. Table S10: GEO matrix after removing batch effects.

**Supporting Information 2** Figure S1: Scatter plots for the five replicated proteins, illustrating consistent trends between genetic instruments and EM risk.

**Supporting Information 3** Figure S2: Funnel plots used to visually assess pleiotropy.

**Supporting Information 4** Figure S3: Leave‐one‐out analysis, confirming that no single SNP strongly influences the overall estimates.

**Supporting Information 5** Figure S4: Forest plots of individual SNP‐level and pooled estimates for the candidate proteins.

## Data Availability

The original contributions presented in the study are included in the article/supporting material, further inquiries can be directed to the corresponding authors.
