# Supplementary material for: Identifying Novel Biomarkers and Therapeutic Targets for Endometriosis: Integrative Analysis of the Plasma Proteome and Genome
Source: Mediators Inflamm. 2026 May 23;2026:6617402. doi: 10.1155/mi/6617402 (PMC13197833; doi:10.1155/mi/6617402)
Supplement: Supplementary file 3 — Supporting Information 3 Figure S2: Funnel plots used to visually assess pleiotropy. [file MI-2026-6617402-s003.pdf]

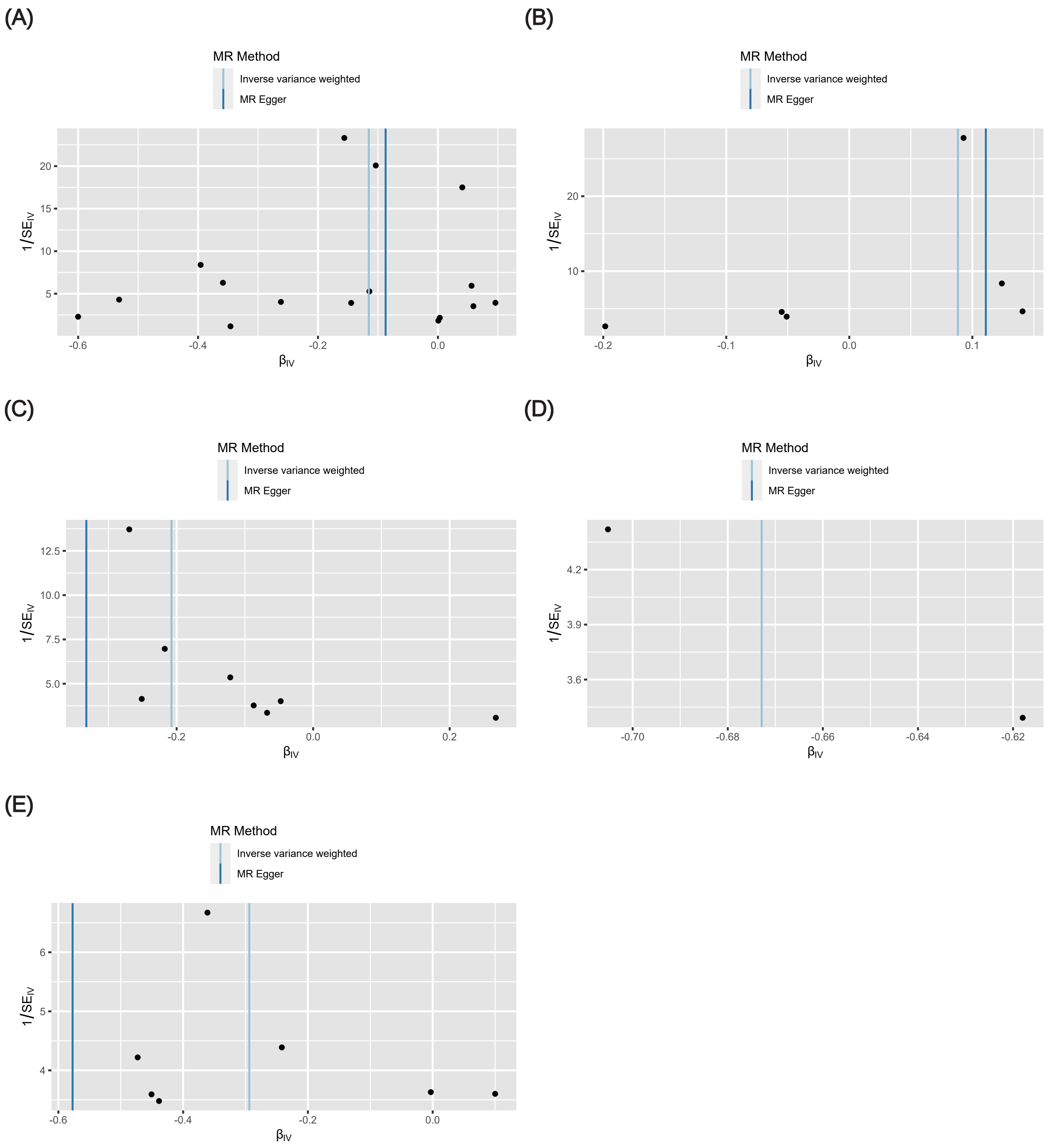

Supplementary File 2 Figure S3. Funnel plot: A. ALPI and EM, B. KHK and EM, C. HSPG2 and EM, D. POLR2F and EM, E. STXBP1and EM.
