# Supplementary material for: Identifying Novel Biomarkers and Therapeutic Targets for Endometriosis: Integrative Analysis of the Plasma Proteome and Genome
Source: Mediators Inflamm. 2026 May 23;2026:6617402. doi: 10.1155/mi/6617402 (PMC13197833; doi:10.1155/mi/6617402)
Supplement: Supplementary file 4 — Supporting Information 4 Figure S3: Leave‐one‐out analysis, confirming that no single SNP strongly influences the overall estimates. [file MI-2026-6617402-s004.pdf]

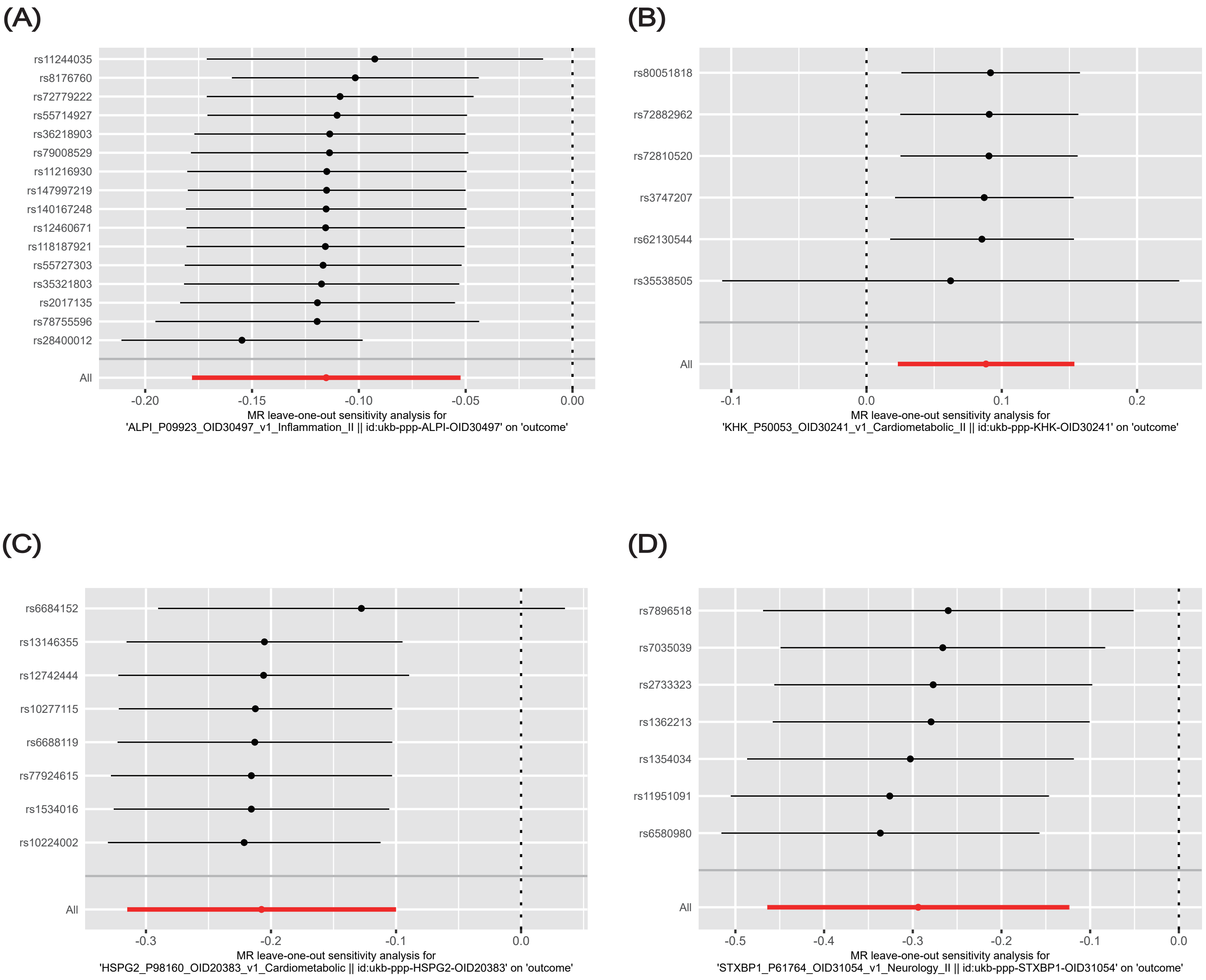

Supplementary File 2 Figure S4. Leave-one-out sensitivity analysis: A. ALPI and EM, B. KHK and EM,
C. HSPG2 and EM, D. STXBP1and EM.
